# Longitudinal study on health-related quality of life and multimorbidity: from trajectories to outcomes in China

**DOI:** 10.3389/fpubh.2025.1643525

**Published:** 2025-10-29

**Authors:** Jiancai Du, Ai Qi, Wenlong Wang, Ximin Ma, Jiahui He, Qi Hu, Kexin Chen, Hui Qiao

**Affiliations:** ^1^School of Public Health, Ningxia Medical University, Yinchuan, Ningxia, China; ^2^Key Laboratory of Environmental Factors and Chronic Disease Control, Ningxia Medical University, Yinchuan, Ningxia, China; ^3^The Center for Disease Control and Prevention in Ningxia Hui Autonomous Region, Yinchuan, Ningxia, China

**Keywords:** quality of life, multimorbidity, longitudinal studies, rural population, China

## Abstract

**Background:**

The association between multimorbidity and health-related quality of life has been extensively studied. This study aimed to investigate the longitudinal association between multimorbidity and health-related quality of life in a rural Chinese population. Specifically, we sought to: (1) examine how the trajectory of multimorbidity burden influences subsequent health-related quality of life, and (2) explore how the trajectory of health-related quality of life, in turn, influences the incidence of multimorbidity.

**Methods:**

Based on a longitudinal survey of the health status of rural community residents aged 18 years and above in 2012, 2015, 2019, and 2022, a group-based trajectory model was used to fit the trajectory of multimorbidity and health-related quality of life utility index over time. Multinomial logistic regression was employed to analyze the association trajectory with outcomes between the health-related quality of life utility index and multimorbidity. A series of sensitivity analyses was used to ensure the robustness of results.

**Results:**

A total of 1,398 residents who completed the four follow-up surveys were included in the analysis. Two trajectories of the health-related quality of life utility index were identified, and the risk of two and three or more multimorbidities in the decline trajectory was 1.95 and 3.78 times greater than that in the stable trajectory, respectively. Two trajectories of multimorbidity were identified: the health-related quality of life utility index decreased by 0.09 in the rapid increase trajectory compared with the slow increase trajectory. The results of the sensitivity analysis remained robust.

**Conclusion:**

The trajectory of the health-related quality of life utility index can be used to predict the risk of multimorbidity. Heterogeneous trajectories of chronic disease multimorbidity development lead to differential impacts on the health-related quality of life utility index.

## 1 Introduction

Multimorbidity, defined by the World Health Organization (WHO), is having two or more chronic diseases simultaneously. A previous systematic review (1) demonstrated that the global prevalence of multimorbidity among adults ranged from 15 to 43% across low-, middle-, and high-income countries based on community-based studies, with a notably higher prevalence of 56.7% observed among adults aged 45 years or older in China (2). A study found that 20%−40% of adults and over half of those aged 65 years and older in the UK had multimorbidity (3). However, multimorbidity is not exclusive to older populations (4); it also significantly affects middle-aged and younger individuals (5). In China, studies have reported the prevalence of multimorbidity was 15.8% (6) in adults aged 30–79 years and 17.6% (7) in those aged 45–59 years. Furthermore, multimorbidity is associated with a range of adverse health outcomes, including reduced quality of life (8), elevated healthcare expenditure (9, 10), and increased the risk of disability (11) and death (6). Given its widespread impact, multimorbidity has emerged as a critical public health challenge worldwide (4).

Given multimorbidity widespread impact on population health, understanding how multimorbidity affects patient-reported outcomes, particularly health-related quality of life (HRQoL), is a critical importance. HRQoL reflects an individual's overall perception of their health status, including physical, social, and psychological health (8, 12). Although the association between multimorbidity and HRQoL has been extensively studied (3, 8, 13), some investigations have shown inconsistent results. Jennifer E. Lutomski's study (14) revealed that multimorbidity patterns are not associated with HRQoL in individuals aged 65+ years in the TOPICS-MDS project. Prior analyses have shown that multimorbidity is associated with a lower quality of life (15–17). One study by Steell et al. (3) demonstrated that multimorbidity clusters are significantly associated with HRQoL in individuals aged ≥18 years from two UK cohorts. Meanwhile, Prior investigations have shown that poorer quality of life can predict adverse health outcomes during follow-up (18). Some studies showed that HRQoL could predict the risk of all-cause mortality in the older adults (19, 20).

However, the aforementioned studies, whether HRQoL or multimorbidity, based on single measurement results, are uncertain and could change over time. It is currently unknown whether HRQoL or multimorbidity change over time and how they relate to one another over time. The studies scarcely use the trajectories of change to study the correlation between multimorbidity and HRQoL. Identifying trajectories considers not only baselines and variations but also long-term trends, provides much more robust results (21). Moreover, identifying trajectories may help with early screening and targeted interventions to reduce the risk of adverse health outcomes (22).

Hence, this current study aimed to examine the longitudinal trajectories of HRQoL and multimorbidity, and to explore their associations in a rural Chinese population.

## 2 Methods

### 2.1 Sample and data collection

The current study used data from the project of innovating a payment system to improve healthcare efficiency, which was conducted by Ningxia Medical University in Northwest China. This project began in 2009 and was followed up every 3–4 years, with more than 20 thousand participants investigated from four counties in Ningxia, northwestern China. This study employed a multistage stratified random sampling method, with participants drawn from community-dwelling permanent residents across four aforementioned counties. The specific sampling methods can be found in the published literature (23). This study utilized four rounds of follow-up data, including waves three (2012), four (2015), five (2019), and six (2022), and included 1,398 respondents. Trained investigators conducted face-to-face interviews in the form of paper questionnaires. The respondents were required to provide written informed consent.

To explore the relationship between the HRQoL and multimorbidity over time, this study included respondents aged ≥18 years who completed four rounds of follow-up measures, and the HRQoL and multimorbidity were included in the current research. A total of 17,768 participants aged ≥18 years completed the interview in 2012 and served as the baseline. The numbers of residents lost to follow-up or death in 2015, 2019, and 2022 were 7,076, 4,198, and 2,197, respectively. In addition, 1,554, 789, 505, and 45 adults were excluded because of the absence of HRQoL in 2012, 2015, 2019, and 2022, respectively. Six respondents were deleted because of inconsistent personal information. Finally, 1,398 adults were enrolled in the analysis.

### 2.2 EQ-5D utility index

The EQ-5D-3L scale, which includes a health description system and a visual analog scale (VAS), is widely used to measure HRQoL in the general population and patients, especially in developing areas and in areas with underdeveloped education levels (16, 24), and has good reliability and validity in the Chinese population (24). The health description system has five dimensions: mobility (MO), self-care (SC), usual activities (UA), pain/discomfort (PD), and anxiety/depression (AD). The current study used the EQ-5D-3L scale to measure the HRQoL of northwestern rural residents. A single item is used to describe each dimension, which is divided into three levels: no problem, moderate problem, and severe problem. We use the Chinese version of the utility rating system (25) to calculate the utility index for each health state, and the specific calculation formula is as follows:


$$\begin{array}{r} EQ-5D-3L\;utility\;index=1-MO2\times 0.0766-MO3\\ \times 0.2268-SC2\times 0.0441-SC3\times 0.2912-UA2\\ \times 0.0370-UA3\times 0.0538-PD2\times 0.0274-PD3\\ \times 0.0409-AD2\times 0.0359-AD3\times 0.1771 \end{array}$$

### 2.3 Multimorbidity

The questionnaire asked the respondents whether they had been diagnosed by doctors with the following 12 chronic diseases: hypertension, diabetes, heart disease, stroke, cancer, intervertebral disc disease, arthritis or rheumatism, epilepsy, severe mental disorder (depression), chronic lung disease, kidney disease, and digestive system disease. Each disease is a binary variable; the answer “yes” is encoded as 1, and “no” is encoded as 0. The number of diseases is equal to the sum of 12 types of disease, which are divided into four categories, namely, 0, 1, 2, and 3 or more. Multimorbidity was defined as having two or more chronic diseases in an individual (16).

### 2.4 Covariates

Given the previous studies (5, 26, 27), this study incorporated covariates such as demographic characteristics, health services, living environment, and economic income. Demographic characteristics comprised sex (0 = male, 1 = female), age group (18–44, 45–59,60) (28), marital status (0 = married, 1 = unmarried, divorced, widowed), and educational level (non-formal, primary school, middle school, high school or more). Health services encompassed medical insurance (0 = yes, 1 = no), outpatient service in the previous 2 weeks (29) (0 = no, 1 = yes), inpatient service in the previous year (0 = no, 1 = yes). Living environment featured the following: housing type (soil-bricked, brick-wooded, soil-wooded, full-bricked, others), type of drinking water (tap-water, well-water, cellar-water, others), having a car (0 = no, 1 = yes), independent kitchen (1 = yes, 2 = no). Economic income covered registered household (0 = no, 1 = yes), previous household income level (1 = < 6,001 Ren Min Bi (RMB), 2 = 6,001–1,0001 RMB, 3 = 10,001–20,001 RMB, 4 = 20,001-RMB).

### 2.5 Statistical analysis

We used the mean and standard deviation to describe continuous variables, absolute numbers and frequency to describe categorical variables. We use the Chi-square test to determine the differences in the composition ratio or rate of categorical variables among different trajectory groups.

We employed GBTM (group-based trajectory modeling), a specialized application of the latent class growth model (30), to explore the possible trajectories of multimorbidity and the EQ-5D utility index. The Stata plugin “traj” was used to estimate group-based trajectory modeling and provide three alternative specifications (31): the censored normal distribution is intended for the analysis of censored continuous scales. The EQ-5D utility index was censored data, with a maximum value of one and a minimum value of zero. Given that the number of chronic diseases in an individual was longitudinal count data, a zero-inflated Poisson (ZIP) distribution was adopted to model the number of multimorbidity diseases. Trajectory models were fitted with two to four groups. For each candidate trajectory, polynomial orders were tested from higher-degree (cubic) to lower-degree terms. Higher-order terms were progressively removed if statistically non-significant (*p* ≥ 0.05), until the most parsimonious intercept-only model (zero-order) was reached (32). This iterative process was repeated for all candidate trajectories to identify statistically viable polynomial specifications. The final model selection was then conducted by comparing composite criteria across competing trajectory solutions. We evaluated the optimal number of trajectories by the following composite criteria. (i) Bayesian information criterion (BIC), where the smaller the BIC value is, the better the fitting effect. (ii) Average posterior probability (AvePP), where the AvePP of each trajectory class is greater than 0.7 (33). (iii) The sample of each trajectory group is not < 5% of the total sample (34).

Multinomial logistic regression was employed to analyze the associations between the trajectory of EQ-5D utility index and multimorbidity with different numbers of chronic diseases in 2022. Tobit regression was used to analyze the associations between the trajectory of multimorbidity and EQ-5D utility index in 2022. The results of the regression analysis were presented as relative risk ratio (RRR), coefficient (Coef), and 95% confidence intervals (95% CI). We constructed three models to adjust for covariates to test the robustness of the results. Model 1 adjusted for age group alone. Model 2 adjusted for sex, marital status, education, and medical insurance. Model 3 further adjusted for smoking, drinking status, and outpatient service in the previous 2 weeks, inpatient service in the previous year, household type, type of drinking water, having a car, independent kitchen, registered household, and household income levels.

There are 10 individuals with missing educational level information, so we use mode interpolation. Lack of smoking, alcohol consumption information at baseline. To address potential bias from missing historical data that might affect multimorbidity assessments, we retrospectively estimated 2012 smoking and alcohol consumption status using prospectively collected 2022 data. The 2022 survey collected detailed smoking/drinking information, including current smoking/drinking status, age at initiation for both substances, and years since quitting. Smoking status for 2012 was determined by calculating smoking duration (2022 age minus smoking initiation age). Participants with ≥10 years of smoking duration were classified as smokers, while those with < 10 years were classified as non-smokers. Additionally, participants who had quit smoking for ≥10 years by 2022 were categorized as non-smokers, whereas those with < 10 years of cessation were considered smokers. The same method was applied to estimate 2012 alcohol consumption status.

To ensure the robustness of the research results, we conducted a series of sensitivity analyses, including removing 10 individuals with missing educational levels; deleting 32 individuals with multimorbidity at baseline; and further adjusting for smoking and alcohol consumption in Model 3.

## 3 Results

### 3.1 Characteristics of the respondents

A total of 1,398 respondents aged 18 years and more were included in the study. The demographic characteristics (age, sex), socioeconomic variables (marital status, educational level, health insurance, household income, housing type, type of drinking water), etc., shows in Tables 1, 2. In our study, there were 699 females (50%), the mean age was 48.1 years at the baseline of 2012, and 1,343 (96.1%) were married. In terms of educational level, 435 (31.1%) had non-formal education, 591 (42.3%) had primary school, 307 (22.0%) had middle school, and 65 (4.6%) had high school and above. The EQ-5D utility index was 0.96 (SD = 0.10), and the prevalence of multimorbidity was 16.1% at the follow-up endpoint of 2022.

**Table 1:** Differences in the demographic characteristics of EQ-5D utility index trajectories.

**Variable**	**Sub-variable**	**Number (*n* = 1,398)**	**EQ-5D trajectories**			
			**Stable (*****n*** = **1,278)**	**Decline (*****n*** = **120)**	*X*^2^/*z*	***P*** **-value**
Sex	Male	699	634	65	0.92	0.340
	Female	699	644	55		
Age-group	18–44 years	574	561	13	124.84	< 0.001
	45–59 years	559	519	40		
	60- years	265	198	67		
Married	Married	1,343	1,234	109	9.51	0.002
	Other	55	44	11		
Educational level	Non-formal	435	373	62	37.49	< 0.001
	Primary school	591	541	50		
	Middle school	307	300	7		
	High school^*^	65	64	1		
Insurance	Yes	1,379	1,260	119	0.27	0.914
	No	19	18	1		
Smoking	No	1,104	1,004	100	1.5	0.22
	Yes	294	274	20		
Alcohol consumption	No	1,303	1,186	117	3.82	0.051
	Yes	95	92	3		
Outpatient in last 2 weeks	No	1,044	969	75	10.30	0.001
	Yes	354	309	45		
Inpatient in last year	No	1,248	1,162	86	42.47	< 0.001
	Yes	150	116	34		
Multimorbidity	No	1,366	1,257	109	27.76	< 0.001
	Yes	32	21	11		
House condition	Brick-soiled	320	296	24	2.64	0.62
	Brick-wooded	459	421	38		
	Soil-wooded	313	280	33		
	Full-bricked	226	206	20		
	Others	80	75	5		
Drink type	Tap	354	332	22	5.01	0.171
	Well	389	358	31		
	Cell	590	530	60		
	Others	65	58	7		
Car	No	1,214	1,107	107	0.62	0.43
	Yes	184	171	13		
Independent kitchen	No	912	834	78	0	0.955
	Yes	486	444	42		
Registered house	No	1,042	968	74	11.45	0.001
	Yes	356	310	46		
Incoming level	−6,001 RMB	372	308	64	53.62	< 0.001
	6,001–10,001 RMB	331	303	28		
	10,001–20,001 RMB	380	365	15		
	20,001- RMB	315	302	13		

^*^Given that the number of individuals with high school education in the EQ-5D trajectory group is relatively small, we combine those with middle and high school education levels for analysis. RMB, Ren Min Bi, the China's legal tender.

**Table 2:** Differences in the demographic characteristics of multimorbidity trajectories.

**Variable**	**Sub-variable**	**Number (*n* = 1,398)**	**Multimorbidity trajectories**			
			**Slow**	**Rapid**	*X*^2^/*z*	***P*** **-value**
Sex	Male	699	425	274	7.9	0.005
	Female	699	373	326		
Age-group	18–44 years	574	455	119	212.61	< 0.001
	45–59 years	559	260	299		
	60- years	265	83	182		
Married	Married	1,343	775	568	5.44	0.02
	Other	55	23	32		
Educational level	Non-formal	435	201	234	49.83	< 0.001
	Primary school	591	335	256		
	Middle school	307	221	86		
	High school^*^	65	41	24		
Insurance	Yes	1,379	787	592	0.01	0.943
	No	19	11	8		
Smoking	No	1,104	611	493	6.47	0.011
	Yes	294	187	107		
Alcohol consumption	No	1,303	729	574	10.07	0.002
	Yes	95	69	26		
Outpatient in last 2 weeks	No	1,044	637	407	26.04	< 0.001
	Yes	354	161	193		
Inpatient in last year	No	1,248	745	503	32.44	< 0.001
	Yes	150	53	97		
EQ-5D	Mean (standard variation)	0.97 (0.09)	0.98 (0.07)	0.95 (0.10)	8.99	< 0.001
House condition	Brick-soiled	320	182	138	4.37	0.358
	Brick-wooded	459	279	180		
	Soil-wooded	313	170	143		
	Full-bricked	226	124	102		
	Others	80	43	37		
Drink type	Tap	354	203	151	2.31	0.51
	Well	389	215	174		
	Cell	590	347	243		
	Others	65	33	32		
Car	No	1,214	684	530	2.06	0.152
	Yes	184	114	70		
Independent kitchen	No	912	525	387	0.25	0.616
	Yes	486	273	213		
Registered house	No	1,042	616	426	6.92	0.009
	Yes	356	182	174		
Incoming level	−6,001 RMB	372	165	207	35.06	< 0.001
	6,001–10,001 RMB	331	195	136		
	10,001–20,001 RMB	380	239	141		
	20,001- RMB	315	199	116		

^*^Given that the number of individuals with high school education in the EQ-5D trajectory group is relatively small, we combine those with middle and high school education levels for analysis. RMB, Ren Min Bi, the China's legal tender.

### 3.2 Trajectories of the EQ-5D utility index and the number of multimorbidity

**Figure 1:**
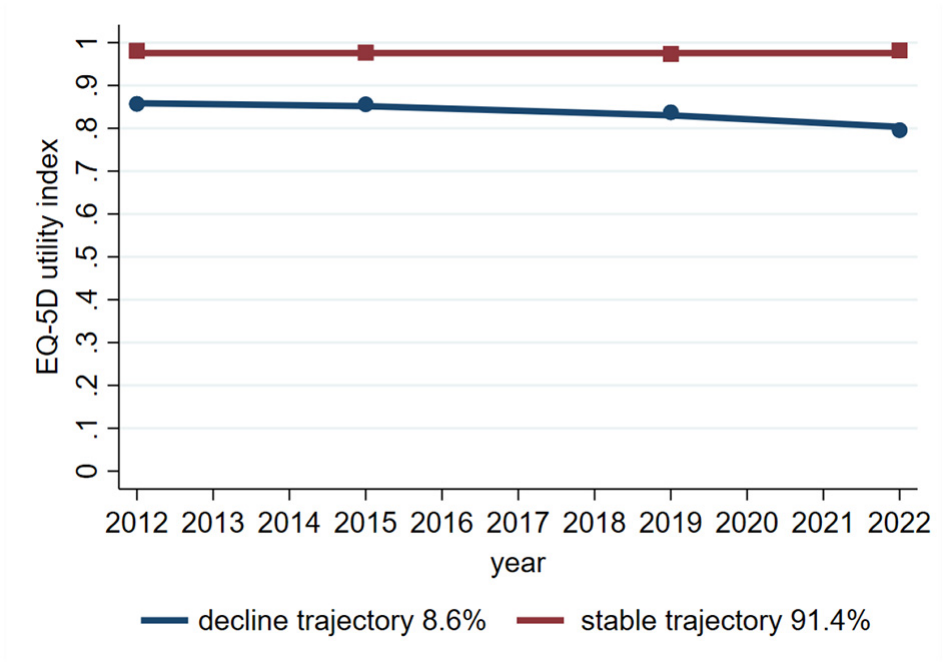
Line graph showing EQ-5D utility index trajectories from 2012 to 2022. The stable trajectory, in red, remains near 1.0, representing 91.4% of cases. The decline trajectory, in blue, decreases slightly over time, representing 8.6% of cases.

Table 3 shows that, based on the BIC criterion and three model evaluation parameters, we ultimately selected two trajectories of the EQ-5D utility index as the optimal trajectory. Figure 1 shows that one characterized by maintaining a stable utility index, which accounted for 91.4% of the total sample, and the other characterized by a gradual declining trend in the utility index, which accounted for 8.6%. Compared with the stable utility index group, there were more people aged 60 years and above, accounting for 56% of the sample; the proportion of people with education levels below primary school accounted for 96%; and the percentage of subjects with 2 weeks of outpatient, prior year of inpatient, and registered household education was greater in the declining trend group. The specific trajectory parameters shows in Supplementary Table 1.

**Table 3:** Model fitting parameters for the trajectories of EQ-5D utility index.

**Trajectory**	**Model**	**BIC**	**Entropy**	**Group membership**	**AvePP**
2	(3 0)	−1,470.49	0.84	8.50/91.50	0.86/0.97
3	(1 2 2)	−1,428.00	0.71	2.00/18.80/79.20	0.87/0.78/0.89
4	(1 1 3 0)	−1,405.16	0.70	1.10/1.90/32.70/64.30	0.91/0.92/0.78/0.83

AvePP, average posterior probability; BIC, Bayesian information criterion.

**Figure 2:**
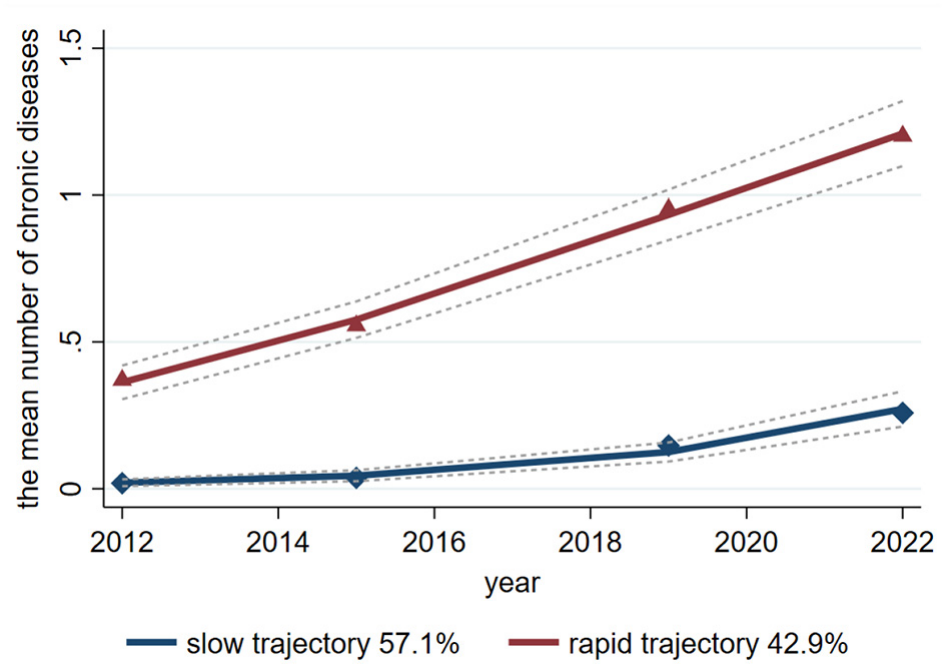
Line graph showing the mean number of chronic diseases from 2012 to 2022 for two trajectories: slow (57.1%) and rapid (42.9%). The slow trajectory rises gradually, while the rapid trajectory increases steeply, with both showing projected trends.

Table 4 shows that, based on the BIC criterion and three model evaluation parameters, two trajectories of multimorbidity were ultimately selected as the optimal trajectories. Figure 2 shows that one characterized by maintaining a slowly increasing trend, which accounted for 57.1%, and the other trajectory showing a rapidly increasing trend, which accounted for 42.9%. In the rapid increasing trajectory, females accounted for 54%, those aged 45 years and above accounted for 80%, and those with educational levels below primary school accounted for 82%. The percentage of subjects with 2 weeks outpatient visits, prior year inpatient visits, and registered household education were greater than that those in the slow increasing trajectory. The specific trajectory parameters shows in Supplementary Table 2.

**Table 4:** Model fitting parameters for the mean number of multimorbidity trajectory.

**Trajectory**	**Model**	**BIC**	**Entropy**	**Group membership**	**AvePP**
2	(1 2)	−4,159.10	0.55	57.1/42.9	0.88/0.86
3	(0 1 0)	−4,183.95	0.67	38.2/58.2/3.6	0.79/0.92/0.73
4	(0 2 0 1)	−4,159.72	0.55	38.2/29.8/0.1/31.9	0.60/0.75/0.58/0.80

AvePP, average posterior probability; BIC, Bayesian information criterion.

### 3.3 Associations of the trajectories of the EQ-5D utility index with the mean number of multimorbidity in 2022

Table 5 shows that compared with the stable group, the decrease in the EQ-5D utility index was associated with a greater risk of having two (RRR = 2.95; 95% CI = 1.63–5.34) and three or more chronic diseases (RRR = 4.78; 95% CI = 2.24–10.19) adjusted covariates on the basis of Model 3. The results of the sensitivity analysis were consistent with the main conclusions, Supplementary Table 3.

**Table 5:** Association of the trajectory for the EQ-5D utility index with the number of chronic diseases.

**Number of chronic disease**	**Model 1**		**Model 2**		**Model 3**	
	**RRR**	**95% CI**	**RRR**	**95% CI**	**RRR**	**95% CI**
0 (reference)	1		1		1	
1	1.73^*^	1.02–2.92	1.66	0.98–2.82	1.63	0.95–2.80
2	3.52^***^	1.99–6.24	3.36^***^	1.88–6.01	2.95^***^	1.63–5.34
3	6.01^***^	2.93–12.31	5.35^***^	2.57–11.12	4.78^***^	2.24–10.19

Model 1 adjusts for the variables of age group; Model 2 adjusts for the variables of sex, marital status, education, and insurance; Model 3 further adjusts for the variables of outpatient service in the previous 2 weeks, inpatient service in the previous year, housing type, type of drinking water, car, independent kitchen, registered household, and previous household income level. RRR, relative risk ratio; CI, confidence interval. *, **, ***, mean *p* < 0.05, *p* < 0.01, and *p* < 0.001, respectively.

### 3.4 The associations of the mean number of multimorbidity trajectory with the EQ-5D utility index in 2022

Table 6 shows that compared with the slow increase trajectory, the rapid increase trajectory in the mean number of multimorbidity was a risk factor for the EQ-5D utility index (Coef = −0.09; 95% CI = −0.13 to −0.06) adjusted for covariates in Model 3. The results of the sensitivity analysis were consistent with the main conclusions, Supplementary Table 4.

**Table 6:** Association of the trajectory of the number of multimorbidity with the EQ-5D utility index.

**Multimorbidity trajectory**	**Model 1**		**Model 2**		**Model 3**	
	**Coef**	**95% CI**	**Coef**	**95% CI**	**Coef**	**95% CI**
Slow increase	1		1		1	
Rapid increase	−0.11^*^	−0.15 to −0.08	−0.11	−0.14 to −0.07	−0.09	−0.13 to 0.06

Model 1 adjusts for the variables of age group; Model 2 adjusts for the variables of sex, marital status, education, and insurance; Model 3 further adjusts for the variables of outpatient service in the previous 2 weeks, inpatient service in the previous year, housing type, type of drinking water, car, independent kitchen, registered household, and previous household income level. Coef, coefficient, CI, confidence interval. *, **, ***, mean *p* < 0.05, *p* < 0.01, and *p* < 0.001, respectively.

## 4 Discussion

This study examines the relationship between the trajectory of EQ-5D utility index and multimorbidity, and the association between the trajectory of multimorbidity and EQ-5D utility index using a representative sample of northwest rural residents in China. In this study, the prevalence of multimorbidity was 16.1%, and the EQ-5D utility index was 0.96 in 2022. The risk of developing two and three or more multimorbidity in the group with a declining EQ-5D utility index trajectory was 2.89 and 5.01 times greater than that in the stable group, respectively. The EQ-5D utility index in the rapidly increasing multimorbidity trajectory group decreased by 0.09 compared with that in the slowly increasing trajectory group.

This study revealed that the prevalence of multimorbidity was 16.1%. Soren T. Skou T. Skou's analyse (1) revealed that the prevalence of multimorbidity in adults worldwide was 15%−43%. A systematic review (7) revealed that the prevalence of multimorbidity in adults in rural areas and western regions in China were 18.3 and 19.6%, respectively. Geng et al. (35) reported that the prevalence of multimorbidity was 46.5% in adults with 12 chronic diseases via self-report, physical examinations and blood sample testing in 2018. Another investigation (36) reported that the prevalence was 5.9% in adults with 15 chronic diseases in 2021. The prevalence of multimorbidity based on self-reports seriously underestimates the true situation, as early detection and management of multimorbidity has become the top priority for multimorbidity prevention and control.

In this study, we used a group-based trajectory model to identify the trajectory of EQ-5D utility index and multimorbidity. The trajectory model considers the baseline level, variability, and trends over time, providing more robust conclusion (21). We identified two trajectories of EQ-5D utility index consistent with Pamela Tanguay's (37) research, which also identified two trajectories of EQ-5D utility index in COVID-19 outpatients aged over 18 years, namely, the stable group and the declining group. A previous analyse (38) identified three trajectories of EQ-5D utility index in road trauma survivors aged ≥18 years, namely, low/moderate-stable, high-large decline, and high-slight decline. HRQoL is heterogeneous among different populations, with the majority of people having relatively stable HRQoL and some experiencing changes, resulting in different trajectories. Compared with the stable trajectory of EQ-5D utility index, the risk incidence of multimorbidity was 2.89 times greater for two chronic diseases and 5.01 times greater for three or more chronic diseases in the decline trajectory. Nearly a quarter of older adults, one-fifth of unmarried individuals (divorced or widowed), and one-seventh of informal education individuals belong to the decline trajectory of EQ-5D utility index. The longitudinal investigation by Mank et al. (39) revealed a significantly steeper decline in EQ-5D utility index measures among dementia patients relative to those with subjective cognitive decline or mild cognitive impairment, establishing the trajectory of EQ-5D utility index as a clinically relevant metric for monitoring therapeutic interventions in Alzheimer's disease. Therefore, declining health-related quality of life may help identify individuals at risk of multimorbidity, especially in rural settings in China and low-resource contexts.

The EQ-5D scale includes five dimensions item and more easily capture some clinical symptoms, which may be non-specific clinical manifestations of undiagnosed chronic diseases. The diagnosis of chronic diseases has obvious hysteresis, especially in rural areas. For example, the awareness of hypertension and diabetes among adults in rural China was 37.6% (40) and 32.6% (41), respectively. Numerous research have shown that multimorbidity was associated with HRQoL, multimorbidity can reduce EQ-5D utility index (3, 12, 42), and conversely, EQ-5D utility index could reflect the presence of multimorbidity to some extent.

In this study, we identified two multimorbidity trajectories, namely, slow growth and rapid growth. Our research results were inconsistent with those in the literature. Hui Chen's analyse (30) identified four multimorbidity trajectories, namely, no new conditions, slow growth, stable growth, and rapid growth, on the basis of the Health and Retirement Study of individuals aged 50 years or above in China. A research (43) identified three multimorbidity trajectories, namely, consistently slow, abrupt increase, and progressive, on the basis of the National Long-Term Care Insurance of older adults in Korea. Another investigation (44) identified four multimorbidity trajectories, namely, mild multimorbidity late progress, mild multimorbidity early progression, moderate multimorbidity, and severe multimorbidity, based on 67 common chronic diseases aged 40 years or older in Sweden. The selection of the target population, the number of diseases included in the analysis, and different countries may all be reasons for inconsistent classification of multimorbidity trajectories. Compared with the multimorbidity trajectory of slow growth, EQ-5D utility index in 2022 was poorer during rapid growth, which aligned with the findings of a previous study (16), which indicated that per additional multimorbidity count was associated with poor EQ-5D utility index.

Multimorbidity reduced the EQ-5D utility index of residents, which was consistent with the findings of literature studies (12, 15). Sai Zhen Sim's analyse showed that participants with three or more conditions had a lower EQ-5D utility index compared to those with one or two condition, aged 40–64 years in Singapore (45). This current research showed that the rapid growth trajectory of multimorbidity, i.e., with more chronic diseases, was associated with a more significant decline in the EQ-5D utility index.

The relationship between multimorbidity and HRQoL can be explained through two key mechanisms. First, HRQoL may reflect early physical or psychological discomfort, which itself could stem from an undiagnosed underlying disease—potentially due to limitations in personal health awareness or healthcare access. Second, diagnosed chronic conditions typically present with noticeable symptoms or clinical signs, contributing to varying degrees of physical or psychological distress and consequently reducing EQ-5D utility index (8, 46). This study found that, after adjusting for covariates, multimorbidity trajectories were associated with declined HRQoL, while changes trajectories of EQ-5D utility index could predict the onset of multimorbidity. However, unmeasured covariates such as physical activity, BMI, and waist circumference may introduce bias into the results, which merits further investigation in future research (47, 48).

Our findings are useful for policy makers who are dedicated to identify and detect multimorbidity for rural adults, particular for those who have not given sufficient attention to unawareness of chronic disease or multimorbidity. The EQ-5D-3L scale is the most widely used instrument (8), renowned for its ease of operation and cost effectiveness. When combined with clinical examinations after preliminary screening using the EQ-5D-3L scale, it is a more effective method to identify patients with multimorbidity. This approach facilitates timely intervention, and minimizes both mortality and the economic burden of diseases associated with multimorbidity.

The strength of this study is that it is the first to use the trajectory of changes in HRQoL to predict the risk of multimorbidity. Second, the study focuses on the trajectory of HRQoL and multimorbidity in individuals aged 18 and above and investigates their related relationships.

Some limitations of this research need to be noted. First, the prevalence of chronic diseases is self-reported, possibly underestimating the prevalence of multimorbidity. Second, among the chronic diseases included in the analysis, hyperlipidemia was not included, which may have further underestimated the prevalence of multimorbidity. Third, the aging of participants during the 10-year follow-up may itself have influenced changes in HRQoL, independently of multimorbidity, suggesting the need for future research to model age as a time-dependent factor or to stratify analyses by age group. Fourth, we used data on smoking and alcohol consumption status from 2022, considered the age of initiation and years since quitting for smoking or alcohol consumption, and estimated their status in 2012. It may introduce bias into the results, but it will not affect the overall trend. Fifth, the EQ-5D-3L scale was more widely used in 2012, whereas the EQ-5D-5L had not yet been widely adopted in China. Although the EQ-5D-5L has a lower ceiling effect, its response distribution in the general Chinese population does not demonstrate a more concentrated pattern compared to the EQ-5D-3L (49). However, influenced by traditional Chinese culture, residents tend to report that their physical condition is better, which may lead to an overestimation of HRQoL, especially in Asian populations (50), and also affects the classification of HRQoL trajectories. Given the loss or death of the follow-up population, only 1,398 people were ultimately included in the analysis sample. By comparing the baseline demographic characteristics between the included and excluded populations, differences were observed in all indicators except for gender, medical insurance, having car, or EQ-5D utility index. Therefore, caution should be taken in the process of extrapolating conclusions. This conclusion needs to be validated in a larger sample population.

## 5 Conclusion

This study revealed that the trajectory of the EQ-5D utility index can predict the risk of multimorbidity, which can be used to screen high-risk populations for multimorbidity. These findings may be relevant for ruralsettings in China and potentially for other low-resource contexts. Identifying multimorbidity through the EQ-5D and incorporating it into management can improve health outcomes such as quality of life and life expectancy. Furthermore, this study verified that different trajectories of multimorbidity can lead to different EQ-5D utility index.

## Data Availability

The raw data supporting the conclusions of this article will be made available by the authors, without undue reservation.
