# Supplementary material for: Longitudinal study on health-related quality of life and multimorbidity: from trajectories to outcomes in China
Source: Front Public Health. 2025 Oct 29;13:1643525. doi: 10.3389/fpubh.2025.1643525 (PMC12605071; doi:10.3389/fpubh.2025.1643525)
Supplement: Supplementary file 1 [file Table_1.docx]

**Supplementary material**

**Longitudinal Study on Health-Related Quality of Life and Multimorbidity: From Trajectories to Outcomes in China Supplementary table 1** Fit parameters of 2 trajectories for the EQ-5D utility index

| Group | Parameter | Estimate | standard Error | *T* | *P* |
| --- | --- | --- | --- | --- | --- |
| 1 | Intercept | 0.90 | 2359.57 | 0.00 | 1.00 |
|  | Linear | -1.11 | 2.52 | -0.44 | 0.66 |
|  | Quadratic | 0.00 | 0.00 | 1.50 | 0.13 |
|  | Cubic | 0.00 | 0.00 | -3.93 | 0.00 |
| 2 | Intercept | 1.13 | 0.01 | 171.31 | 0.00 |
|  | Sigma | 0.18 | 0.00 | 53.52 | 0.00 |

**Supplementary table 2** Fit parameters of 2 trajectories of multimorbidity

| Group | Parameter | Estimate | standard Error | *T* | *P* |
| --- | --- | --- | --- | --- | --- |
| 1 | Intercept | -2.60 | 0.17 | -15.30 | 0.00 |
|  | Linear | 2.59 | 0.28 | 9.11 | 0.00 |
| 2 | Intercept | -0.29 | 0.05 | -5.45 | 0.00 |
|  | Linear | 1.21 | 0.08 | 15.73 | 0.00 |
|  | Quadratic | -0.48 | 0.24 | -2.02 | 0.04 |

**Supplementary table** 3 Association of the trajectory of the EQ-5D utility index with the number of multimorbidity

| Number multimorbidity | RRR | Standard  Error | *Z* | *P* | 95%CI | |
| --- | --- | --- | --- | --- | --- | --- |
|  |  |  |  |  | LL | UL |
| 0(refer) |  |  |  |  |  |  |
| 1* | 1.69 | 0.47 | 1.89 | 0.06 | 0.98 | 2.92 |
| 2* | 3.07*** | 0.94 | 3.67 | 0.00 | 1.69 | 5.60 |
| 3* | 4.61*** | 1.81 | 3.88 | 0.00 | 2.13 | 9.97 |
| 1# | 1.53 | 0.43 | 1.51 | 0.13 | 0.88 | 2.65 |
| 2# | 2.70*** | 0.84 | 3.18 | 0.00 | 1.46 | 4.97 |
| 3# | 4.78*** | 1.94 | 3.85 | 0.00 | 2.16 | 10.61 |
| 1& | 1.57 | 0.45 | 1.59 | 0.11 | 0.90 | 2.75 |
| 2& | 2.74 | 0.87 | 3.19 | 0.00 | 1.47 | 5.09 |
| 3& | 4.77 | 2.00 | 3.73 | 0.00 | 2.10 | 10.84 |

Attention: * indicates that 10 individuals were deleted because of missing education levels. # indicates that 32 individuals were excluded because of multimorbidity in 2012. & indicates that 42 individuals were excluded because of missing education levels or multimorbidity in 2012, and further adjustments were made for the covariates of smoking and alcohol consumption based on Model 3. RRR, relative risk ratio; CI, confidence interval. LL, lower limit; UL, upper limit.

**Supplementary table 4** Association of the trajectory of the number of multimorbidity with the EQ-5D utility index

| Trajectory multimorbidity | Coef | Standard  Error | *T* | *P* | 95%CI | |
| --- | --- | --- | --- | --- | --- | --- |
|  |  |  |  |  | LL | UL |
| Slow increase |  |  |  |  |  |  |
| Rapid increase* | -0.09 | 0.02 | -5.30 | 0.00 | -0.13 | -0.06 |
| Rapid increase# | -0.09 | 0.02 | -5.29 | 0.00 | -0.13 | -0.06 |

Attention: * indicates that 10 individuals were excluded because of missing educational levels. # indicates further adjusted covariates of smoking and alcohol consumption based on Model 3. Coef, coefficient, CI, confidence interval. LL, lower limit; UL, upper limit.

**Supplementary Table 5** Demographic differences between deleted and included participants

| Variable | Sub-variable | Number  (n=17768) | Deleted data  (n=16370) | Analysis data  (n=120) | $\chi^{2}/Z$ | *P* |
| --- | --- | --- | --- | --- | --- | --- |
| Sex | Male | 9156 | 8457 | 699 | 1.42 | 0.233 |
|  | Female | 8612 | 7913 | 699 |  |  |
| Age-group | 18-44 years | 10576 | 10002 | 574 | 249.71 | <0.001 |
|  | 45-59 years | 4301 | 3742 | 559 |  |  |
|  | 60- years | 2891 | 2626 | 265 |  |  |
| Married | Married | 13618 | 12275 | 1343 | 319.75 | <0.001 |
|  | Other | 4150 | 4095 | 55 |  |  |
| Educational level | Non-formal | 4606 | 4171 | 435 | 127.79 | <0.001 |
|  | Primary school | 6055 | 5474 | 581 |  |  |
|  | Middle school | 4817 | 4510 | 307 |  |  |
|  | High school | 2184 | 2119 | 65 |  |  |
|  | missing | 106 | 96 | 10 |  |  |
| Insurance | Yes | 17433 | 16054 | 1379 | 0.09 | 0.768 |
|  | Non | 225 | 206 | 19 |  |  |
| Outpatient in last 2 weeks | Non | 2916 | 2562 | 354 | 87.82 | <0.001 |
|  | Yes | 14852 | 13808 | 1044 |  |  |
| Inpatient in last year | Non | 1582 | 1432 | 150 | 6.24 | 0.013 |
|  | Yes | 16186 | 14938 | 1248 |  |  |
| Multimorbidity | Non | 17556 | 16190 | 1366 | 15.46 | <0.001 |
|  | Yes | 212 | 180 | 32 |  |  |
| House condition | Brick-soiled | 4377 | 4057 | 320 | 21.89 | <0.001 |
|  | Brick-wooded | 5581 | 5122 | 459 |  |  |
|  | Soil-wooded | 4318 | 4005 | 313 |  |  |
|  | Full-bricked | 2834 | 2608 | 226 |  |  |
|  | Others | 658 | 578 | 80 |  |  |
| Drink type | Tap | 4007 | 3653 | 354 | 16.59 | 0.001 |
|  | Well | 5022 | 4633 | 389 |  |  |
|  | Cell | 7509 | 6919 | 590 |  |  |
|  | Others | 1230 | 1165 | 65 |  |  |
| Car | Non | 15469 | 14255 | 1214 | 0.07 | 0.796 |
|  | Yes | 2299 | 2115 | 184 |  |  |
| Independent kitchen | Non | 10902 | 9990 | 912 | 9.63 | 0.002 |
|  | Yes | 6866 | 6380 | 486 |  |  |
| Registered house | Non | 12815 | 11773 | 1042 | 4.39 | 0.036 |
|  | Yes | 4953 | 4597 | 356 |  |  |
| Incoming level | 0-6001 RMB | 4323 | 3951 | 372 | 17.77 | <0.001 |
|  | 6001-10001 RMB | 3786 | 3455 | 331 |  |  |
|  | 10001-20001 RMB | 4891 | 4511 | 380 |  |  |
|  | 20001- RMB | 4768 | 4453 | 315 |  |  |
| EQ-5D* | mean(standard variation) | 0.97  (0.09) | 0.97  (0.09) | 0.97  (0.08) | 1.49 | 0.137 |

Attention: RMB, Ren Ming Bi, the China's legal tender. * Only 9174 respondents completed the EQ-5D scale assessment
